# Production of Glucoamylase from Novel Strain of *Alternaria Alternata* under Solid State Fermentation

**DOI:** 10.1155/2022/2943790

**Published:** 2022-10-27

**Authors:** Durr-e- Nayab, Shamim Akhtar, Nazneen Bangash, Waqar-un- Nisa, Malik Tahir Hayat, Awais Zulfiqar, Mubashar Niaz, Abdul Qayyum, Asad Syed, Ali H. Bahkali, Abdallah M. Elgorban

**Affiliations:** ^1^Department of Botany, University of Gujrat, Pakistan; ^2^Department of Biosciences, COMSATS University, Islamabad, Pakistan; ^3^International Islamic University, H-10 Islamabad, Pakistan; ^4^Department of Environmental Sciences, COMSATS University, Abbottabad Campus, Islamabad, Pakistan; ^5^Brimbank City Council, P.O. Box 70 Sunshine, Victoria 3020, Australia; ^6^Atlas Environmental Laboratories, Suite 1503, Street 36 West, Manhattan, New York 10018, USA; ^7^Department of Agronomy, The University of Haripur, Haripur 22620, Pakistan; ^8^Department of Botany and Microbiology, College of Science, King Saud University, P.O. 2455, Riyadh 11451, Saudi Arabia

## Abstract

Glucoamylase has an essential role as biocatalyst in various important industries of Pakistan. It is synthesized by using various fungal and bacterial strains, and different ecocultural conditions are applied under solid substrate fermentation method (SSF) to get the highest yield of glucoamylase. *Alternaria alternata* is an important fungus that can grow on industrial raw material like wheat bran, dried potato powder, tea leaves, rice husk, and sugar cane peel which are used as substrate. Among all, dried potato powder (10g) proved the best fermentation media for growth of fungal strain as well as maximum glucoamylase producer. Moreover, several chemical and physical states were also explored through solid substrate fermentation technique on glucoamylase yield. The highest glucoamylase production was recorded after 72 hours incubation in incubation chamber with 10g raw substrate, 1ml inoculum spore solution, 30°C temperature, and 5 pH. Further, phosphate buffer (5 pH) as moistening agent, 5% starch concentration and media additive as nitrogen (yeast extract), and carbon source (maltose) were screened for maximum glucoamylase titer (17.3 ± 0.05^a^°U/ml/min) and the highest specific activity (39.2U/mg). These cultural conditions were most appropriate for growth of *A. alternata* on solid media and production of highest glucoamylase under solid state fermentation procedure that could be utilized for commercial synthesis of glucoamylase.

## 1. Introduction

Enzymes are globular proteins that are used to speed up the various biochemical reactions. All the metabolic reactions are catalyzed due to the presence of enzymes in the body. There are a lot of enzymes that have attributes at industrial levels, such as in paper sizing, fabric, pharmaceutical, soap, and fermentation industry. Glucoamylase (*α*-1,4-glucan glucohydrolase, amyloglucosidase, EC 3.2.1.3) as a catalyst is used in food industry, textile industry, and detergent industry to mediate the industrial process without being used in the whole practice [1]. Glucoamylase has the ability to convert starch into the end product of *β*-D-glucose by breaking 1-4 glycosidic or 1-6 glycosidic linkages from non-reducing ends of oligo and polysaccharides chains. This multidomain glycoprotein comprises of about 640 amino acids. The larger catalytic domain (CD), that is, the N-terminal part of the enzyme, contains 1-470 amino acids while the starch binding domain, that is, C-terminal part, having 509-640 amino acids. Both domains of glucoamylase are linked by a linker region that has 471-508 amino acids. There is a cleft in starch binding domain where the substrate is strongly attached, but this domain has no role in the catalytic activity of glucoamylase. The catalytic domain catalyzes the breakdown of *α*-1, 4-, and *α*-1, 6-glycosidic linkages of the substrate to produce free glucose residues. The linker region also has no role in the catalytic activity of the glucoamylase, but it provides the structural integrity to the enzyme [2].

The sources of glucoamylase are animals, plants, and microorganisms [3]. Microbes like yeast, bacteria, and fungi are the cheapest source to produce glucoamylase [4]. The process is called microbial hydrolysis, and it is carried out on industrial wastes by these microbes. The microbes are also used to break down the by-products of agriculture for the manufacturing of many compounds such as hormones, various enzymes, and organic acids [5]. Now, industrial biotechnology focuses on various fungal sources include *Aspergillus awamori*, *Rhizopus delmar*, *A*. *oryza*, *A. niger*, *Neurospora*, and *Mucor rouxians* for glucoamylase production [6].

The substrate of fungus *A. alternata* may be plants, soils, and food items. *A. alternata* has many catalytic activities in various industries for the manufacturing of different kinds of end products. Many enzymes are being produced on industrial scale due to their high demands. Similarly, solid substrate fermentation mechanism has been used for enzyme production. The growth of microbes on solid material in presence of excess water is called submerged fermentation, and microbial growth on moist solid stuff without excess water is called solid substrate mechanism. Almost all type of enzyme production can be obtained by solid state fermentation, and it is an advantage over the submerged fermentation due to its easy and less expensive methodology [7].

Solid state mechanism can be used for the synthesis of many enzymes on large industrial scale at very low cost. Different solid media as rice bran, potato starch granules, bagasse powder, wheat bran, and tea waste is used through the solid state fermentation [8]. For maximum glucoamylase synthesis, a variety of substrates are used. After screening of substrate, optimum conditions such as substrate concentration, inoculum level, and optimum incubation period are applied to attain maximum glucoamylase production. Optimum pH and temperature are another essential factor to get maximum glucoamylase synthesis. Similarly, buffers and distilled water are important moistening agents, which are used for maximum synthesis of glucoamylase [9]. Various other factors are also used to enhance the production of glucoamylase. These factors include optimization of additional media as carbon sources and various nitrogen sources. These all are being studied as essential requirements to enhance the rate of glucoamylase [10].

The most important role of glucoamylase is the breakdown of starch into small units of glucose. The glucose is used at industrial scale such as in food industries and beverage industry. It also plays a key role as an important substrate in the process of fermentation and used for the growth of microbial strains. As glucoamylase is an essential industrial enzymes, so various microorganisms involved in production of glucoamylase are under investigation. Many researches also indicate that fungal and bacterial strains have the ability to meet the universal needs of metabolites and enzymes under solid state fermentation than submerged state fermentation [11]. Due to unfortunate economy of Pakistan, glucoamylase is being imported from other countries, which are rich in its production to meet its dire need and demands. Glucoamylase as biocatalyst is linked to the production of essential industrial products, so it is important to optimize conditions for its production. The production of glucoamylase from waste material could be a novel approach for its synthesis and utilization.

## 2. Materials and Methods

### 2.1. Isolation and Maintenance of Cultural Strain

Fungal culture was obtained from rotten tomato and maintained in the Mycology and Biotechnology Laboratory, GCU Faisalabad on 10 ml autoclaved potato dextrose agar media (potato infusion 200 ml, 20 g dextrose, and 20 g Agar). The experiment was repeated for purification of fungi [12]. The isolated fungal culture was named by using a code MBL-T, where MBL represents the Mycology and Biotechnology Laboratory and T represenst Tomato, as the strains were isolated from tomato.

### 2.2. Identification of Fungal Strain

Fungal culture was examined by carrying the fungal hyphae on sterilized glass slide containing Lactophenol cotton blue in 70% alcohol. The prepared slide was examined under the microscope (LABOMED Model: Lx 400) with camera (LABOMED, iVu 1500). Morphological data of fungal mycelium and reproductive structure was obtained by using different lens (10X, 40X, etc.). Identification was done by using standard literature and web sources as MycoBank. Different fungal attributes were studied for cultural strain identification like shape and color of conidiophores, fungus colony, sporangia, conidia as well as hyphal morphology, etc.

### 2.3. Mechanism of Fermentation

For biosynthesis of glucoamylase, a measuring flask of 250 ml was used which has moisture of about 9 ml and substrate of about 10 g. Flasks were cooled after sterilization at 121°C and 15 pressure for 2 hours. Spore suspension of 1 ml (4.42 × 10^−7^ spores) was added in all flasks containing raw substrate (10 g dried potato powder) under aseptic condition [13]. The flasks were placed in an incubator for 72 hours at 30°C.This experiment was performed in triplicate [14].

### 2.4. Enzyme Extraction

For enzyme extraction, after 72 hours fermentation, 100 ml of distilled water was added, and mixture was kept in rotary shaker for an hour at 30°C and 150 rpm. After that, it was filtered through Whatman filter paper No.1 and transferred to 250 ml flasks. The filtrate was used to estimate the activity of glucoamylase [15].

### 2.5. Glucoamylase Assay

All test tubes containing 1 ml enzyme extract and 1 ml of 5% starch solution were kept in water bath for one hour at 60°C. The test tubes were cooled after adding 2 ml of DNS reagent. After cooling, all these tubes were placed in boiling water for 5 minutes to stop the reaction. Test tubes were cooled at room temperature. For dilution, distilled water of 16 ml was poured in each tube. The absorbance was measured by spectrophotometer at 546 nm absorbance. Experimental and control value was measured. Glucoamylase units and glucoamylase activity were done by subtracting the control value from experimental value [16].

#### 2.5.1. Glucoamylase Unit

Enzyme assay is a condition in which 1 unit is the quantity of enzyme that releases 1 micromole glucose. by consumption of substrate per min per mL [15].

#### 2.5.2. Glucoamylase Activity

The glucoamylase activity was obtained by applying the formula, which is given below [9].

(1) $$\begin{array}{c} \text{Glucoamylase activity}\left(\text{U}/\text{mL}/\min\right)=\frac{\text{Glucose obtained}}{180\times 60}\times 1000. \end{array}$$

### 2.6. Assay for Protein Analysis

For protein determination, 1 ml enzyme extract and 5 ml Bradford solution were poured in each trial tube. After 10 minutes, 9 ml distilled water was flowed in each experimental tube. Then, absorbance was evaluated at 595 nm from the standard curve of BSA, and a relationship was derived to determine the protein. By the use of same amount of distilled water, control solution was also estimated in the other test tube, and then it was deducted from the experimental determinations [14].

#### 2.6.1. Specific Activity

Specific activity was measured by using formula.

(2) $$\begin{array}{c} \text{Specific Activity}\left(\frac{\text{U}}{\text{mg}}\right)=\frac{\text{Activity of Enzyme}}{\text{Total Protein}\left(\text{mg}/\text{mL}\right)}. \end{array}$$

### 2.7. Ecocultural Environment for Maximum Enzyme Yield

Various cultural conditions were studied to optimize the production of glucoamylase from *A. alternata* by using the solid media as substrate.

#### 2.7.1. Substrates Used

Dried potato powder, used tea leaves, rice brain, wheat bran, and sugarcane peel were used as substrate to optimize the glucoamylase yield [17].

#### 2.7.2. Different Weight of Substrate

Different concentrations of optimized substrates like 5 g, 10 g, 20 g, and 25 g (w/v) were examined for optimization of the production of glucoamylase. These concentrations of screened substrates were taken in sterilized flask of 250 ml, and after 72 hours, enzyme activity was determined as reported by [18].

#### 2.7.3. Incubation Time

The incubation period of 24 hours, 48 hours, 72 hours, 96 hours, and 120 hours was used to attain the maximum production of glucoamylase under previous optimized cultural conditions [19].

#### 2.7.4. Optimization of Spore Suspension

To study the highest yield of glucoamylase different inoculum levels, such as 1 ml, 1.5 ml, 2 ml, 2.5 ml, and 5 ml, with different amount of spore size was optimized [13].

#### 2.7.5. Optimization of Incubation Temperature

To enhance the glucoamylase value, a range of incubation temperatures like 20°C, 30°C, 40°C, 50°C, and 60°C was optimized under former optimized conditions for fungal growth and enzyme production [9].

#### 2.7.6. pH Range Optimization

For highest production of glucoamylase, optimum pH range is required. For this purpose, screened solid media was taken in Erlenmeyer flask of 250 ml under former optimized culture conditions, and then, it was suspended in the buffers of aqueous solution of different pH like 3, 4, 5, 6, and 7 [20].

#### 2.7.7. Optimization of Starch Concentration

Different concentrations of starch such as 2 ml, 3 ml, 4 ml, 5 ml, and 6 ml [21] were used to study the increase in glucoamylase production.

#### 2.7.8. Moisture Level Optimization

Phosphate buffer, acetate buffer, tap water, distilled water, and mineral water are some moistening agents. These moistening agents with pH 5 were used to record the increase in production of glucoamylase [22].

#### 2.7.9. Optimization of Various Media Additives

Urea, yeast extract, trypton, soya bean, meal, and NH_4_NO_3_ are some nitrogen sources, and glucose, maltose, galactose, lactose, and sucrose are various carbon sources. These all were applied to solid medium as additional media additives to increase the glucoamylase titer in preoptimized conditions [22].

### 2.8. Statistical Analysis

The data was analyzed by using the computer software cohort costat. All experiment was conducted in triplicate, and average value of enzyme activity was reported. Duncan's multiple range test (DMRT) was applied under one way Anova. Minitab software was used for the principle component analysis (PCA).

## 3. Results and Discussion

### 3.1. Identification of Fungal Strain as *Alternaria Alternata* (MBL-T)

In this study, the identification of glucoamylase producing fungal strain was done by using related internet sources as MycoBank, standard literature, and monographs that based on its morphological study and cultural attributes. According to this study, the isolated fungal strain showed black to greyish color with dark brown conidiophores that contain asexual conidiospores (conidia) that exhibited the resemblance with *Alternaria alternata* that was codified as MBL-T.

### 3.2. Effect of Various Solid Substrates

**Figure 1:**
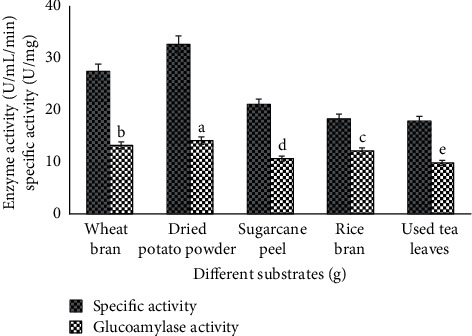
Comparison of glucoamylase production and specific activity by using various raw media for growth of *A. alternata.*

For industrial economy, glucoamylase is a commercial enzyme that is obtained from diverse fungal sources. *A. alternata* (MBL-T) is the vital and cheapest microbial source of glucoamylase. The effects of many physical and chemical factors were examined under solid substrate fermentation to get maximum glucoamylase yield by using *A. alternata* (Figure 1). Rice bran, tea waste, sugarcane peel, dried potato powder, and wheat bran were used as fermentation media [17]. These agroindustrial substances were used as raw material to grow the fungal strain for highest production of glucoamylase. Dried potato powders (specific activity 32.7 U/mg and glucoamylase activity 14.1 ± 0.19^*a*^ U/ml/min) were screened among all other substrates for the better glucoamylase yield. Potato culture media proved to be the best substrate for glucoamylase production for the growth of *Rhizopus oryzae*. So, the results revealed that the use of potato as diffusion juices with agar and sugar or with wheat bran was applied in biotechnology field for the growth of various fungal strains [23], but the maximum yield of glucoamylase by using dried potato powder as a fermentation media under solid state fermentation is a novel phenomenon in which *Alternaria alternata* was grown on this solid substrate.

### 3.3. Effect of Different Levels of Substrate and Inoculum

**Figure 2:**
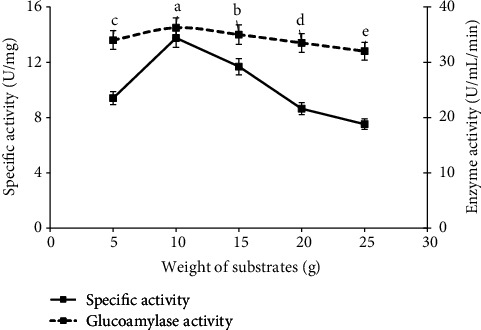
Study of glucoamylase production and its specific activity on different weight of raw media by growth of *A. alternata.*

Similarly, the media weight such as 5, 10, 15, 20, and 25 g with 1 ml (4.42 × 10^−7^) inoculum level was used, and the solid media of 10 g (specific activity 34.4 U/mg and glucoamylase activity 14.5 ± 0.09a U/ml/min) (Figure 2) was screened as best substrate of fungal growth and enzyme activity due to availability of sufficient oxygen at this substrate level. Similarly, inoculum level of 0.5, 1, 1.5, 2, and 2.5 ml was used to study the fungal growth and enzyme production with spore count of 2.21 × 10^−7^, 4.42 × 10^−7^, 6.72 × 10^−7^, 9.27 × 10^−7^, and 11.43 × 10^−7^ spores, respectively, and the 1 ml (4.42 × 10^−7^ spores) of inoculum level among different levels of spore suspension showed the maximum specific activity of 36.0 U/mg and glucoamylase units of 15.5 ± 0.09^*a*^ U/ml/min) (Figure 2) in the presence of selected media (10 g). In case of substrate concentrations, these results were parallel with the findings of [4, 24] as the adequate amount of media was necessary for the maximum enzyme rate. Their results determined that the very low concentration of substrate is inefficient for growth of fungal strains due to less available nutrients and surface area for the growth of fungi and microbial enzymes to act upon it while more substrate thickness (higher amount of substrate) showed improper aeration and inadequate agitation that affects the aerobic conditions for fungal growth and decrease the enzyme production. Similarly, optimized level of spore suspension is necessary to get maximum enzyme yield and biomass production. So, this study revealed that negative correlation between enzyme production and higher fungal amount and the study was similar as of [25, 26]. According to this study, the less amount of inoculum level needs more time for fermentation procedure as well as the higher level of inoculum will increase the moisture that leads toward the gas transfer limitations, coagulation of fungal strains, and increase the competition of fungal strains for growth, nutrition, and metabolic process that ultimately decrease the enzyme production.

**Figure 3:**
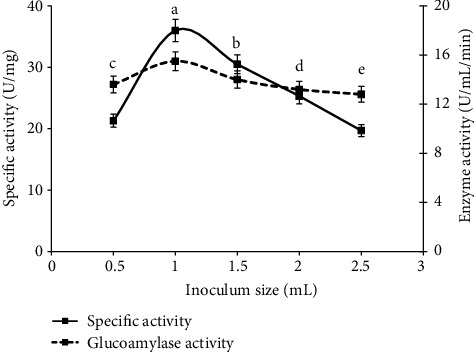
Comparison of glucoamylase activity and specific activity on different level of spore suspension under solid state fermentation.

Glucoamylase activity and specific activity on different level of spore suspension under solid state fermentation are shown in Figure 3.

### 3.4. Effect of Incubation Period

**Figure 4:**
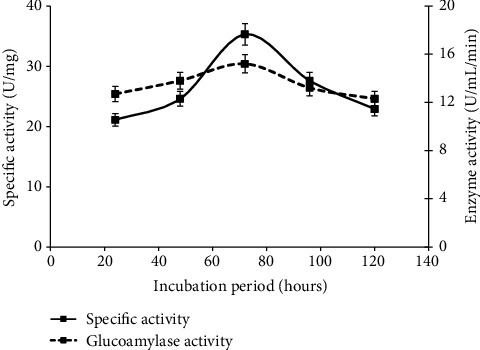
Evaluation of glucoamylase production and its specific activity on different incubation period under solid state mechanism.

Different time period of 24, 48, 72, 96, and 120 hours was studied to determine incubation interval of the highest glucoamylase yield, and it was noted that 72 hours' incubation period (specific activity U/mg and 14.5 ± 0.05^*a*^ U/ml/min) was best for further proceedings (Figure 4). These results are similar to the findings of [9], as the enzyme production rates vary in different microorganisms, and it also depends on media composition and type. Fermentation rate of 48 to 70 hours was determined in most of the fungal species such as *Saccharomyces* and *Aspergillus* species. So, in current study, the glucoamylase yield declined after the fermentation time of 72 hours due to less amount of media nutrients utilized by fungal plugs of *A. alternata*. In short, glucoamylase yield decreased at less incubation period because of minimum enzyme activity, and it was also low at the highest fermentation period because of the formation of products that are toxic and due to insufficient nutrition [15].

### 3.5. Effect of Incubation Temperature

**Figure 5:**
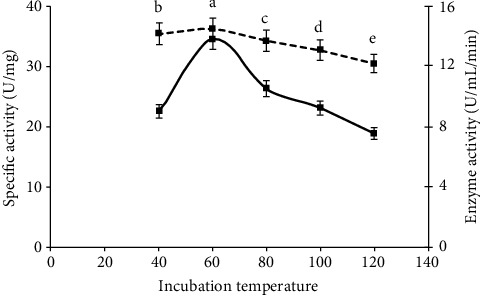
Use of different incubation temperature to study the highest yield of glucoamylase and its specific activity under solid state condition.

Gradually increasing temperature of 30°C was optimized for better glucoamylase production and fungal growth by solid state fermentation (Figure 5). These results had similarity with the findings of [22]. They reported that most of the fungal strains give higher production of enzymes between 30°C to 40°C, and some have maximum production at 60°C. For the adequate production of enzyme, optimum temperature is required because protein structure of enzymes is hydrolyzed, and enzymes lose its activity at very high temperature due to formation of heat in experimental media. So, the optimum temperature for glucoamylase was measured at 30°C to 40°C in previous experiments [22].

### 3.6. Effect of pH

**Figure 6:**
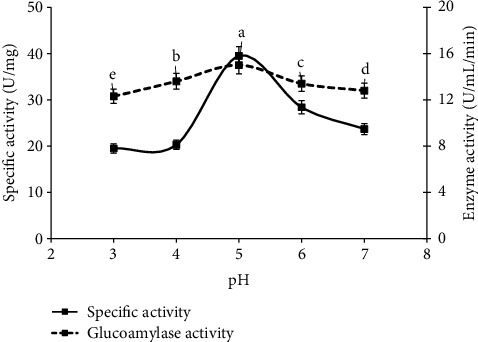
Study of glucoamylase yield and specific activity on different pH value by using the previous screened conditions under solid state mechanism.

Similarity, to increase the enzyme value, pH range of 3 to 7 was also observed under former selected conditions, and pH of 5 (specific activity 34.6 U/mg and glucoamylase value 15.0 ± 0.09^a^ U/ml/min) was optimized for maximum glucoamylase yield. This could be related to the relative stability of glucoamylase at pH 5 (Figure 6). These results were in line with the results of [4] as concentration of pH effect the growth of fungal strains and enzyme production was more adequate in acidic medium. In short, change in the pH level of media that is necessary for growth of fungal strains was directly related to the metabolic activity of enzyme. Hence, enzymes become inactive at inadequate pH level. So, the optimum pH is required for the proper growth of fungi and to get the highest yield of enzyme [4].

### 3.7. Effect of Moistening Agents

**Figure 7:**
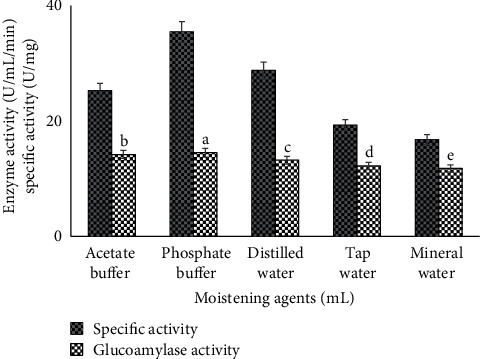
Comparison of glucoamylase production and specific activity by using various moisture contents for growth of *A. alternata.*

Different moisture levels such as tap, distilled, and mineral water as well as phosphate and acetate buffers were also studied to measure the growth of fungi and its effect on glucoamylase production. All moisture levels were used with pH 5 as the substrate for fungal growth, and enzyme production was optimized at pH 5. It was observed that phosphate buffer with pH 5 (35.5 specific activity U/mg and 14.5 ± 0.10^*a*^ U/ml/min enzyme activity) act as best moistening agent (Figure 7). These results were in parallel of [8]. They revealed that high quantity moisture level decreased the fungal growth by reduction in porosity of substrate particles and insufficient oxygen transfer. Similarly, less or no moisture contents also reduced the fungal growth and fermentation process. So, the results could be due to the reason that phosphate buffer with additional media supports the adequate oxygen, porosity temperature, and aeration to fermentation media.

### 3.8. Effect of Starch Concentration

**Figure 8:**
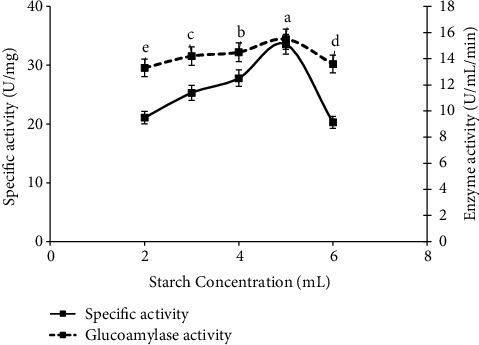
Optimization of glucoamylase production and specific activity by using different concentration of starch by *A. alternata.*

Concentration of starch as 2%, 3%, 4%, 5%, and 6% was also optimized to get high titer of glucoamylase by applying the previous screened cultural conditions that was the use of 10 g dried potato powder with 1 ml inoculum level in the presence of phosphate buffer (pH 5) and incubated for 72 hours at 30°C. Starch concentration (5%) (specific activity 33.6 U/mg and enzyme activity 15.5 ± 0.09^a^ U/ml/min) was selected by using *A. alternata* in solid fermentation media mechanism (Figure 8). The results had similarity with the finding of [22] as the yield of glucoamylase was affected by different concentrations, and 4% starch gave maximum glucoamylase units from *Aspergillus fumigats* than the lower starch level, and it varied from organism to organism. So, the results showed that highest amount of starch concentration was more suitable for maximum glucoamylase yield than lower value of starch by *A. alternata* [22].

### 3.9. Effect of Nitrogen and Carbon as Substrate Additives

**Figure 9:**
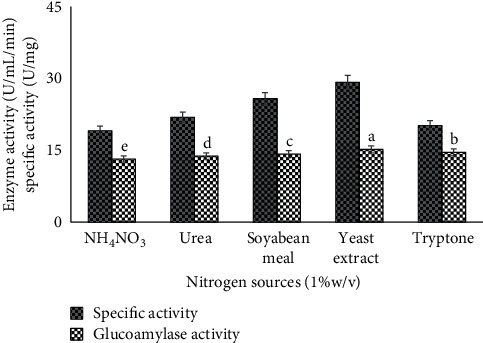
Use of different nitrogen additives to study the glucoamylase production and specific activity under solid state fermentation by *A. alternata.*

**Figure 10:**
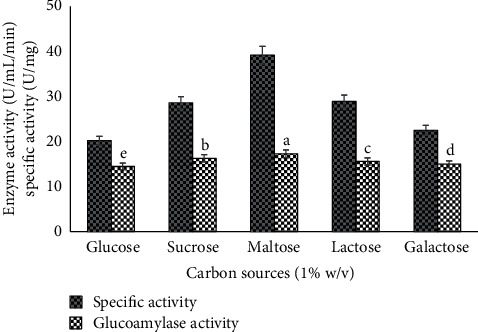
Use of different nitrogen additives to study the glucoamylase production and specific activity under solid state fermentation by *A. alternata.*

Glucoamylase has also a remarkable impact on its yield by applying simple and complex nitrogen and carbon sources as nutritional media with substrate, which are used by fungal strain for its growth and production of glucoamylase. The maximum enzyme production probably depends on microbial demand of *C* and *N* availability by adding simple and complex resources in combination. To increase the production of GA, different nutritional media additives were also optimized like carbon supplies (glucose, galactose, lactose, sucrose, and maltose) and nitrogen additives (urea, tryptone, yeast extract, soybean meal, and NH_4_NO_3_). Yeast extract (Figure 9) and maltose (Figure 10) give the highest units of glucoamylase (specific activity 39.2 U/mg and 17.3 ± 0.05^*a*^ U/ml/min enzyme activity) as the best nutrient rich media for glucoamylase production and fungal growth. These results were corresponding to [22]. They described that different fungal strains have different glucoamylase production rate with carbon or nitrogen sources. Some *Aspergillus* species inhibit enzyme production in presence of glucose, and some *Rhizopus* species decrease glucoamylase yield in presence of fructose. They also reported the yeast extract and urea as nitrogen source for maximum glucoamylase titer by *Rhizopus* species. The results revealed that nutrients dependent enzyme as glucoamylase was constrained by the availability of simple nutrients resources. It was noted that, the presence of assimilable resources may decrease the production of glucoamylase due to inadequate growth of fungal strains. So, the additional media nutrition in even complex form increased the enzyme production by fulfilling the growth requirement of *Alternaria alternata*. The results of glucoamylase production from maltose and yeast extract by *A. alternata* (MBL-T) are a novel approach.

### 3.10. PCA Analysis

**Figure 11:**
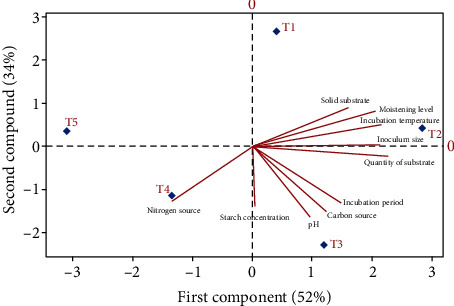
Principal component analysis (PCA) and loading plot-derived correlation between different ecocultural conditions for glucoamylase production by using *A. alternata* through solid substrate fermentation and partial least squares-discriminant analysis (PLS-DA) score plot indicated the variation between different treatments of ecocultural parameters to optimize the best treatment among all.

**Figure 12:**
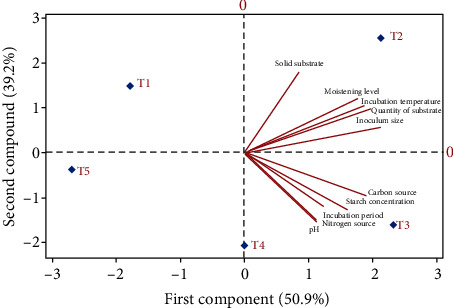
Principal component analysis (PCA) and loading plot-derived correlation between different ecocultural conditions to determine specific activity by using *A. alternata* through solid substrate fermentation and partial least squares-discriminant analysis (PLS-DA) score plot indicated the variation between different treatments of ecocultural parameters to optimize the best treatment among all.

This study evaluated the relationship between various ecocultural conditions of solid state fermentation and their treatment levels by using principal component analysis (PCA). The results were observed in biplot axes, which showed the interaction between variables and treatments. The biplot of the two PC's provided information regarding the correlation between measured properties/parameter and variation between five different treatments in each parameter. It exhibited different parameters association, solid substrate, quantity of substrate, inoculum level, pH, incubation temperature, moistening agents, starch concentration, incubation period, and nitrogen and carbon sources that showed maximum range of variation as well as the study of interaction between these variables that influenced by different treatments. The distance between the locations of any two treatments is directly proportional to the degree of difference or similarity between them that is showed by scores on biplot, and the properties/parameters with curve lines that are close to each other showed the positively correlation (Figures 11 and 12). The results indicated that pH, carbon source, starch concentration, incubation period, and quantity of substrate were positively correlated, and nitrogen sources are negative with each other while solid substrate inoculum size and incubation temperature revealed the weak correlation shown in (Figure 11), similarly. All ecocultural parameters showed positive correlation in determination of specific activity of enzyme (Figure 12). Overall, each treatment in all parameter/ecocultural conditions was different from each other in terms of their negative and positive scores. The T1, T2, and T3 in first component were positively correlated, and T4 and T5 in second component were negatively correlated, while the T1 showed the weak correlation (Figure 11). Further, protein estimation determined the positive correlation between T2 and T4 and negative correlation between T1 and T5, while T3 showed weak correlation (Figure 12). The first principal component (PC's) revealed the eigenvalues > 1 represented to 52.4% for glucoamylase activity and 50.9 for specific activity. Similarly, the second component (PC's) showed the eigenvalues > 1 denoted 34.7% for glucoamylase production and 39.2% for specific activity of the variance.

So, with the proper growth of fungus *A. alternata* under the technique of solid substrate fermentation (SSF), there are many other aspects which are important for glucoamylase production. These aspects are optimized cultural conditions as adequate use of agroindustrial waste, screening of substrate, some substrate concentrations, inoculum level and incubation temperatures, incubation time, pH range, moistening agents, starch concentration, and carbon and nitrogen sources [4]. In the absence of these important aspects, the production and yield of glucoamylase would be low. These all factors must be optimized for glucoamylase yield because of its important application in industries as they are considered the most important [7], and they have positive or negative correlation among themselves as well as among treatments levels [27].

## 4. Conclusion

The present study shows that the isolated fungal strain of *Alternaria alternata* (MBL-T) from rotten tomato has the potential to produce glucoamylase as an important biocatalyst that can be used in various industrial processes. It was concluded that *A. alternata* isolated from rotten tomato has inherent properties for highest glucoamylase production of 17.3 ± 0.05^*a*^ U/ml/min, and the highest specific activity of 39.2 U/mg under optimized conditions as 10 g dried potato powder were used as fermentation media with 1 ml (4.42 × 10^−^7) inoculum level at 30°C temperature and 5 pH. After 72 hours incubation, use of buffer (5 pH) as moistening agent, starch concentration of 5%, and media additive as nitrogen (yeast extract) and carbon source (maltose) increased glucoamylase titer under solid state fermentation, that can increase the glucoamylase activity to meet the industrial needs by using kitchen waste.

## Data Availability

All data related to this research work is included in figures.

## References

[1] Meng S., Yin Y., Yu L. (2019). Exploration of a high-efficiency and low-cost technique for maximizing the glucoamylase production from food waste. *RSC Advances*.

[2] Riaz M., Rashid M. H., Sawyer L. (2013). Physiochemical properties and kinetics of glucoamylase produced from deoxy- -glucose resistant mutant of Aspergillus niger for soluble starch hydrolysis. *Food Chemistry*.

[3] Ayodeji A. O., Bamidele O. S., Kolawole A. O., Ajele J. O. (2017). Physicochemical and kinetic properties of a high salt tolerant Aspergillus flavus glucoamylase. *Biocatalysis and Agricultural Biotechnology*.

[4] Fadel M., AbdEl-Halim S., Sharada H., Yehia A., Ammar M. (2021). Production of glucoamylase, *α*-amylase and cellulase by Aspergillus oryzae F-923 cultivated on wheat bran under solid state fermentation. *Journal of Advances in Biology & Biotechnology*.

[5] Nuryana I., Perwitasari U., Mawey J., Ngangi J., Moko E., Melliawati R. (2020). The improvement of glucoamylase production by UV irradiated strains of Aspergillus awamori KT-11. *IOP Conference Series: Earth and Environmental Science*.

[6] Adeoyo O. R., Pletschke B. I., Dames J. F. (2018). Purification and characterization of an amyloglucosidase from an ericoid mycorrhizal fungus (Leohumicola incrustata). *AMB Express*.

[7] Balakrishnan M., Jeevarathinam G., Kumar S. K. S., Muniraj I., Uthandi S. (2021). Optimization and scale-up of *α*-amylase production by Aspergillus oryzae using solid-state fermentation of edible oil cakes. *BMC Biotechnology*.

[8] Fernandes H., Moyano F., Castro C. (2021). Solid-state fermented brewer’s spent grain enzymatic extract increases in vitro and in vivo feed digestibility in European seabass. *Scientific Reports*.

[9] Osho M. B., Solomon T. G. (2020). Use of composite agro-substrates for amyloglucosidase synthesis and characterization by Aspergillus niger otf and Aspergillus flavus CLOR1 using solid state fermentation. *Journal of Microbiology, Biotechnology and Food Sciences*.

[10] Kshirsagar S., Waghmare P., Saratale G. (2020). Composition of synthesized cellulolytic enzymes varied with the usage of agricultural substrates and microorganisms. *Applied Biochemistry and Biotechnology*.

[11] Benabda O., M’Hir S., Kasmi M., Mnif W., Hamdi M. (2019). Optimization of protease and amylase production by Rhizopus oryzae cultivated on bread waste using solid-state fermentation. *Journal of Chemistry*.

[12] Vidya C. H., Gnanesh Kumar B. S., Chinmayee C. V., Singh S. A. (2020). Purification, characterization and specificity of a new GH family 35 galactosidase from Aspergillus awamori. *International Journal of Biological Macromolecules*.

[13] Chilakamarry C. R., Mimi Sakinah A. M., Zularisam A. W. (2022). Advances in solid-state fermentation for bioconversion of agricultural wastes to value-added products: opportunities and challenges. *Bioresource Technology*.

[14] Melo F. S., Alves I. D. C., Barbosa T. C. V., Cabral L., Santos T. (2019). Effect of temperature in *Α*-amylase and amyloglucosidase produced from English potato residue via fermentation in solid state. *Journal of the Brazilian Chemical Society*.

[15] Singh R. K., Singh A. K., Kumar Y., Masih H. (2019). Production, optimization and characterization of glucoamylase from agricultural residues using aspergillus Niger. *The Pharma Innovation*.

[16] Tayyab M., Ali H., Muneer B. (2019). Optimization of conditions for the production of glucoamylase from aspergillus fumigatus: purification and kinetic studies of glucoamylase. *Journal of Animal and Plant Sciences*.

[17] Escaramboni B., Garnica B. C., Abe M. M., Palmieri D. A., Fernández Núñez E. G., de Oliva Neto P. (2022). Food waste as a feedstock for fungal biosynthesis of amylases and proteases. *Waste and Biomass Valorization*.

[18] El-Metwally M. M., Mohammed Y. M. M. (2019). Production and application of thermostable glucoamylase from thermotolerant Aspergillus fumigatus via semisolid state fermentation. *Egyptian Journal of Botany*.

[19] Reis N., Lessa O. A., Pacheco C. S. V. (2020). Cocoa shell as a substrate for obtaining endoglucanase and xylanase from Aspergillus oryzae ATCC 10124. *Acta Scientiarum-Technology*.

[20] Akcan N. (2018). Cultural conditions optimization for production of *β*-galactosidase from bacillus licheniformis ATCC 12759 under solid-state fermentation. *Turkish Journal of Biochemistry*.

[21] Mansoor S., Tayyab M., Jawad A. (2018). Refolding of misfolded inclusion bodies of recombinant *α*-amylase: characterization of cobalt activated thermostable *α*-amylase from geobacillus SBS-4S. *Pakistan Journal of Zoology*.

[22] Vandenberghe L. P. S., Pandey A., Carvalho J. C. (2021). Solid-state fermentation technology and innovation for the production of agricultural and animal feed bioproducts. *Systems Microbiology and Biomanufacturing*.

[23] Tang X., Luo T., Li X. (2018). Application and analysis of Rhizopus oryzae mycelia extending characteristic in solid-state fermentation for producing glucoamylase. *Journal of Microbiology and Biotechnology*.

[24] Ahmed El-Gendy M. M. A., Hassan Alzahrani N. (2020). Solid state fermentation of agro-industrial residues for glucoamylase production from endophytic fungi penicillium javanicum of solanum tuberosum L. *Journal of Microbial and Biochemical Technology*.

[25] Batista B. N., Matias R. R., Oliveira R. L., Albuquerque P. M. (2022). Hydrolytic enzyme production from açai palm (Euterpe precatoria) endophytic fungi and characterization of the amylolytic and cellulolytic extracts. *World Journal of Microbiology and Biotechnology*.

[26] Gowthaman M. K., Krishna C., Moo-Young M. (2001). Fungal solid state fermentation - an overview. *Applied Mycology and Biotechnology*.

[27] Huang X., Fan Y., Lu T. (2020). Composition and metabolic functions of the microbiome in fermented grain during light-flavor baijiu fermentation. *Microorganisms*.

